# A Cross-Lagged Study of Developmental Trajectories of Video Game Engagement, Addiction, and Mental Health

**DOI:** 10.3389/fpsyg.2018.02239

**Published:** 2018-11-21

**Authors:** Elfrid Krossbakken, Ståle Pallesen, Rune Aune Mentzoni, Daniel Luke King, Helge Molde, Turi Reiten Finserås, Torbjørn Torsheim

**Affiliations:** ^1^Department of Psychosocial Science, Faculty of Psychology, University of Bergen, Bergen, Norway; ^2^School of Psychology, University of Adelaide, Adelaide, SA, Australia; ^3^Department of Clinical Psychology, Faculty of Bergen, University of Bergen, Bergen, Norway

**Keywords:** internet gaming disorder, gaming disorder, longitudinal study, adolescents, mental health

## Abstract

**Objectives:** Video game addiction has been associated with an array of mental health variables. There is a paucity of longitudinal studies investigating such associations, and studies differentiating addicted gaming from problem and engaged (i.e., frequent but non-problem) gaming. The current explorative study investigate the natural course of gaming behavior in three sub-studies. The aim of study 1 was to investigate antecedents and consequences of video game addiction measured as a unidimensional construct (pathological gaming). Aim of study 2 was to investigate the same associations in terms of typologies of gamers (“engaged,” “problem,” “addicted”). Furthermore, study 3 aimed to investigate the estimated stability and transitions occurring between the aforementioned typologies, and a non-pathological gaming group.

**Methods:** A nationally representative sample of 3,000 adolescents aged 17.5 years was drawn from the population registry of Norway in 2012 and invited to participate in annual surveys spanning 3 years (N^T1^ = 2,059, N^T2^ = 1,334, N^T3^ = 1,277). The respondents completed measures of video game addiction, depression, anxiety, loneliness, aggression, and alcohol use disorder. Statistical analysis comprised cross-lagged path modeling, Satorra-Bentler chi square test (study 1), regression analyses (study 2), hidden Markov model of transition probabilities (study 3).

**Results:** Findings in study 1 showed that depression and loneliness were reciprocally associated with pathological gaming. Physical aggression was identified as an antecedent, and anxiety was a consequence of pathological gaming. Investigation of the three typologies of gamers (study 2) identified loneliness and physical aggression as antecedents, and depression as a consequence of all typologies. Depression was found to be an antecedent of problem and engaged gamers. Loneliness was found as a consequence of problem gamers, and anxiety was a consequence of addicted gamers. High alcohol consumption was found antecedent to addicted gamers, and low alcohol consumption was found antecedent to problem gamers. The estimated stability of video game addiction was 35%.

**Conclusion:** A reciprocal relationship between pathological gaming and measures of mental health problems seems to exist. The stability of video game addiction indicates a condition that for a substantial number of people does not resolve spontaneously over the course of 2 years.

## Introduction

Playing video games is a common pastime activity among adolescents that, for the majority, provides hours of fun, challenge, relaxation and socialization (Hoffman and Nadelson, 2010). However, some individuals report that they lose control over their gaming behavior, resulting in significant functional impairment and distress. The concept of video gaming as an addictive disorder was included as a condition for further study in the fifth and latest version of the Diagnostic and Statistical Manual of Mental Disorders (DSM-5) (American Psychiatric Association, 2013), denoted as “Internet Gaming Disorder (IGD).” Similarly, “Gaming disorder” has been included in the 11th version of the International Classification of Diseases (ICD-11) (World Health Organization, 2018). However, critics of the introduction of a diagnosis for video game addiction have argued that the current evidence base warranting such a diagnosis is not sufficient (Van Rooij and Kardefelt-Winther, 2017) and there is still paucity of studies illuminating the natural course of the disorder (Petry and O'brien, 2013; Mihara and Higuchi, 2017). A major limitation related to most of the existent research on gaming disorder is that it mainly involves cross-sectional designs. Longitudinal studies on this topic are few in number (Gentile et al., 2011; Brunborg et al., 2014; Mihara and Higuchi, 2017), although such studies may help identify factors relating to the temporal order of cause and effect, as well as provide knowledge about temporal stability of gaming behavior. The overall aim of the present explorative study were to obtain a broad understanding of the natural course of gaming behavior through three sub-studies (study1, study2, study3). Study 1 conceptualized pathological gaming as a unidimensional entity and investigated cross lagged associations between pathological gaming and mental health over time. Study 2 explored the associations between mental health and gaming categories using a typological perspective, to further investigate the nature of the associations found in study 1. Study 3 investigated the stability and trajectories over time, applying the typological perspective (engaged, problem and addicted gamers) used in study 2.

## Antecedents and consequences of pathological gaming

Longitudinal studies might investigate whether mental health problems first and foremost are predictors of gaming disorder, whether mental health problems mainly are consequences of gaming disorder, or whether the relationship between mental health problems and gaming disorder are of a cross-lagged nature. Cross-lagged effects estimate the reciprocal relationship between variables over time, describing their mutual influence on each other (Kearney, 2018). Thus, identification of cross-lagged effects between mental health and gaming disorder might elucidate the mechanisms involved in development and maintenance of gaming disorder. Although a few studies on the cross-lagged association between gaming addiction and mental health exist (Lemmens et al., 2011a,b), there is overall a dearth of knowledge concerning the cross-lagged associations with gaming disorder, and different mental health problems in large and representative samples.

Previous research has shown that gaming disorder is associated with an array of health-related and social problems (Wittek et al., 2016; Bargeron and Hormes, 2017), such as depression, anxiety (Mentzoni et al., 2011; Bargeron and Hormes, 2017; Wartberg et al., 2017). loneliness (Lemmens et al., 2011a), alcohol use (van Rooij et al., 2014), and aggression (Kim et al., 2008). One longitudinal study identified both anxiety and depression as consequences of pathological gaming after 2 years (Gentile et al., 2011), and two longitudinal studies identified depression, but not anxiety, as a consequence of gaming disorder after one (van Rooij et al., 2011), and 2 years (Liau et al., 2015), respectively. Regarding potential causes of gaming disorder, one study found that depressive symptoms did not predict future gaming problems (Mößle and Rehbein, 2013). A Dutch longitudinal study found loneliness to be both an antecedent and a consequence of pathological gaming (Lemmens et al., 2011a), indicating that loneliness might be important both for the development and maintenance of gaming addiction. Furthermore, there are several cross-sectional studies with mixed evidence on the association between gaming disorder, aggression, and alcohol consumption. Whilst the effect of violence in video games on real life aggression is debated (Funk et al., 2004; Ferguson, 2015) there are also findings suggesting that gaming disorder, regardless of content, can increase aggression in boys (Lemmens et al., 2011b), and that individuals with aggressive tendencies are more likely than their counterparts to develop pathological gaming (Kim et al., 2008). Some cross-sectional studies have shown alcohol-related problems and gaming disorder to be associated (Ko et al., 2008; van Rooij et al., 2014), other studies have not found such association (Brunborg et al., 2014; Kaess et al., 2017).

Across several studies, sex seems to be a robust predictor of video gaming, as males are more likely to engage in video games (Mentzoni et al., 2011; van Rooij et al., 2014; Yu and Cho, 2016) and to be categorized as problem gamers than females (Mentzoni et al., 2011; Brunborg et al., 2013; Yu and Cho, 2016; Milani et al., 2017). However, there are also studies indicating that sex differences are not relevant regarding antecedents and consequences of gaming (Lemmens et al., 2011a; Brunborg et al., 2013). Still, there is need for more in-depth knowledge concerning sex as a moderator in the pathogenesis of gaming disorder (APA Task Force on Violent Media, 2015), and for longitudinal exploration on the association between mental health and gaming disorder.

### Study 1

#### Cross lagged relationship between mental health and pathological gaming using an unidimensional perspective

Previous longitudinal studies have assessed pathological gaming as a unidimensional construct (Lemmens et al., 2011a; Yu et al., 2015), where the symptoms were collapsed and gaming pathology was measured on a continuum ranging from low to high severity. A unidimensional conceptualization of pathological gaming enables investigation of cross-lagged relationship between symptoms of gaming disorder and mental health in one model, indicating reciprocal relationship between the variables over time (Jeon, 2015).

Against this backdrop, the aim of the first study was to identify antecedents and consequences, as well as sex differences, of video game problems. An unidimensional conceptualization of gaming disorder used in previous studies (Lemmens et al., 2011a; Andreassen et al., 2013), was applied (termed “pathological gaming” in the current study). We expected to find several cross-lagged associations between mental health and symptoms of pathological gaming. Due to the explorative nature of this study, and the mixed evidence from previous studies, all variables (mental health and pathological gaming) were investigated both as antecedents, and as consequences of pathological gaming.

### Study 2

#### Antecedences and consequences of pathological gaming using a typological perspective

The conceptualization of gaming disorder emphasizes functional impairment and psychological distress to distinguish the disorder from high involvement in gaming (Charlton and Danforth, 2007; Brunborg et al., 2013; Kardefelt-Winther et al., 2017). A research challenge in this area has been the identification of variables that clearly differentiate between gaming involvement and unhealthy gaming (problems and disorder). While it has been argued that engagement (i.e., healthy use of games) primarily involves salience, tolerance and mood modification (peripheral criteria), gaming disorder typically involves conflict, withdrawal, relapse and problems due to gaming (core addiction criteria) (Charlton and Danforth, 2007; Brunborg et al., 2015) as well. Gaming problems have typically been defined as satisfying some, but not all of the core addiction criteria (Brunborg et al., 2013; Wittek et al., 2016).

Applying a typological perspective enables investigation of whether there are any similarities or differences between “addicted gamers,” “problem gamers,” and “engaged gamers.” In line with such distinction, one study found gaming engagement to be more weakly associated with mental health outcome than addiction (Loton et al., 2016), and other studies have reported no association between video game engagement and mental health problems (Brunborg et al., 2013, 2014). Identification of such distinctions might be relevant for further comprehension of the natural course of gaming disorder, and development of clinical assessment tools, treatment and prevention strategies. To the best of our knowledge there is no previous study investigating associations between mental health and typologies of different gaming behaviors in a large representative sample of adolescents longitudinally.

The aim of Study 2 was thus to investigate antecedents and consequences of the three typologies (addicted, problem, and engaged) of gamers over time. We expected to find a larger number of antecedents, and a larger number of consequences associated with “addicted gamers,” than for “problem gamers,” and “engaged gamers.” Due to the explorative nature of this study, and lack of previous studies investigating different gaming behaviors applying a typological approach, all variables (mental health and gaming) were investigated both as antecedents and consequences.

### Study 3

#### Temporal stability and developmental trajectories using a typological perspective

In addition to exploration of causes and consequences, longitudinal studies provide the possibility to investigate stability of a condition over time. The temporal stability of gaming disorder provides an indication of whether the disorder is a transient problem that resolves spontaneously, due to for instance maturation, or if the condition is rather persistent. To date, results from studies investigating the stability of gaming disorder have been mixed. One study found a high temporal stability of 84% after 2 years (Gentile et al., 2011), while another found that 50% of heavy online gamers with gaming disorder symptoms, remained stable after 1 year (van Rooij et al., 2011). Other studies have reported stabilities as low as 2.8% after 1 year (Rothmund et al., 2018) and < 1% after 2 years (Strittmatter et al., 2016). To the best of our knowledge, there is no study on the developmental trajectories of different typologies of gamers over time. Hence, trajectories between “addicted gamers,” “problem gamers,” “engaged gamers” and normal/non-gamers have previously not been investigated, although this may shed important light on developmental patterns related to gaming behavior.

Therefore, the aim of study 3 was to investigate the temporal stability of “addicted gamers,” and the transitions occurring between “problem gamers,” “addicted gamers,” and “engaged gamers” over time.

## Methods

### Procedure and sample

All three studies employed data from the same large longitudinal survey of gambling, gaming, and drug behavior in adolescents. A nationally representative sample of 3,000 adolescents (50% female) aged 17.5 years was drawn from the Norwegian Population Registry in 2012 (Wave 1). The adolescents were informed about the purpose of the study, that all data would be treated confidentially, and that data would be used only for research purposes. Written informed consent was obtained from all the participants. Parental consent was not required on account of the adolescents being above the age of 16. All who responded at Wave 1 received annual follow-up surveys by postal mail (2013 and 2014) with up to two reminders for each wave. The survey could be answered on paper and returned via an included prepaid envelope or answered online. All participants received a gift certificate with a value of 200 NOK (~18 UK £) upon completion of each wave. The study, including the consent procedure aforementioned, was approved by Regional Committee for Medical and Health Research, Ethics, South East Region (Project Number: 2012/914).

Data from all three waves (2012, 2013, 2014) were used in the three studies included in the current paper. Of the 3,000 adolescents who were invited in 2012, 54 were not reachable due to invalid addresses, whereas 23 were not able to respond due to other reasons such as cognitive disability, reducing our sample to 2,923. In the first wave, 2,059 adolescents responded (response rate 70.4%, 53% female). Four cases were excluded because they were younger than 17 years old, and four cases did not indicate their sex and were excluded. In the second wave, a total of 1,334 individuals responded (retention rate 64.9%, female 58.7%); and in the final wave, 1,277 responded (retention rate 62.1%, female 61.7%).

### Measures and instruments

#### Demographic variables

The questionnaire contained sociodemographic questions including sex.

#### Gaming

Pathological gaming was assessed using the Game Addiction Scale for Adolescents (GASA) (Lemmens et al., 2009). The GASA contains seven items measured on a five-point scale with response options ranging from “never” (Hoffman and Nadelson, 2010) to “very often” (Petry and O'brien, 2013). A composite score was calculated by adding the score of each item. The scale has also been used for differentiating between engaged, problem, and addicted gamers using the CORE 4 approach (Brunborg et al., 2013, 2015; Wittek et al., 2016), by categorizing the different groups according to Charlton and Danforth's (2007) criteria's for addiction and highly engagement (Charlton and Danforth, 2007). The respondents were categorized as “addicted gamers” when all four of the items measuring core criteria of addictin (relapse, withdrawal, conflict and problems due to gaming) were endorsed, and as a “problem gamer” when two or three core criteria of addiction were endorsed. Adolescents who endorsed all three items considered peripheral to addiction (salience, tolerance, and mood modification) and not more than one of the core criteria of addiction were categorized as “engaged gamers.” The remaining respondents comprised the non-addicted/non-problem/non-engaged contrast group (which also include non-gamers). To differentiate between the dimensional use of GASA and the three typologies, “pathological gaming” will in the following be used as a term when referring to the dimensional use of GASA, whereas “engaged gamer,” “problem gamer,” and “addicted gamers” are used for the typological approach. Cronbach's alpha for GASA at the three waves were 0.89, 0.90, and 0.90, respectively.

#### Anxiety and depression

To measure symptoms of anxiety and depression the Hospital Anxiety and Depression Scale (HADS) (Zigmond and Snaith, 1983) was administered. HADS assesses non-vegetative symptoms of depression and anxiety with seven items assessing anxiety and depression, respectively. All items are answered on a four-point scale ranging from 0 to 3. Higher scores indicate higher symptom severity. Cronbach's alpha for the three waves was 0.76, 0.80, and 0.81 for anxiety, and 0.69, 0.73, and 0.76 for depression, respectively.

#### Loneliness

To measure loneliness we administered the Roberts UCLA Loneliness Scale (RULS) (Roberts et al., 1993). RULS contain eight items assessing loneliness on a four-point scale ranging from “never” (Hoffman and Nadelson, 2010) to “often” (Van Rooij and Kardefelt-Winther, 2017). The respondents are instructed to indicate to which extent each statement applies to them. Cronbach's alpha for this scale were 0.75, 0.81, and 0.80 at Waves 1–3.

#### Alcohol consumption

Alcohol consumption was assessed with the Alcohol Use Disorder Identification Test-Consumption (AUDIT-C) (Bush et al., 1998). AUDIT-C assesses alcohol consumption with three items assessing frequency and quantity of drinking on a five-point scale ranging from 0 to 4. Higher scores on the AUDIT-C indicates higher alcohol consumption. Cronbach's alphas for this scale were 0.77, 0.71, and 0.67 at Waves 1–3.

#### Aggression

The Physical and Verbal aggression subscales of the Short Form Buss-Perry Aggression Questionnaire (BPAQ-SF) (Diamond and Magaletta, 2006) were used to assess these constructs. The Physical and Verbal subscales contain four and three items, each answered on a five-point scale ranging from “very unlike me” (0) to “very like me” (4). A high score indicates higher tendency toward aggression. At Waves 1–3 the Cronbach alphas were 0.80, 76, and 0.78 for the physical aggression subscales, and 0.66, 0.68, and 0.67 for the verbal aggression subscale.

### Statistical analyses

Preliminary analysis and attrition analysis was conducted using SPSS, version 25 (Corp, 2017). For attrition analysis, we constructed a nominal variable reflecting completion of all 7 GASA-items (yes or no). Participation on all waves was categorized as 1, missing at T2 only was categorized as 2, missing at T3 only was categorized as 3, and missing on both T2 and T3 was categorized as 4. We then conducted a multinomial regression analysis using sex, and the following measures on T1 as antecedents: “pathological gaming,” “addicted gamer,” “problem gamer,” “engaged gamer,” depression, anxiety, loneliness, verbal aggression, physical aggression, and alcohol consumption.

Further analysis was carried out using the multi-group path analysis in Mplus, version 7.4 (Muthén and Muthén, 2015).

### Study 1

**Figure 1:**
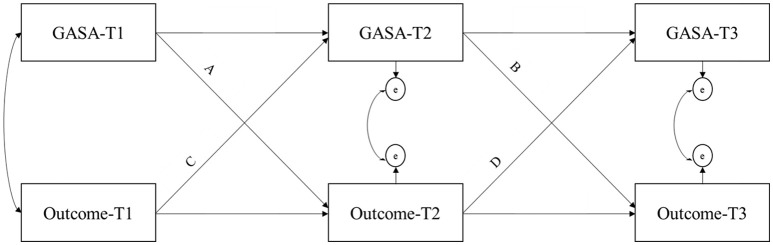
Cross-lagged path modeling of pathological gaming (GASA) against mental health variables (outcome measures).

In the first study, a cross lagged path model with observed indicators was tested to measure the cross-lagged effect of mental health outcomes and gaming across the three waves (see Figure 1). Maximum likelihood estimation with robust standard errors was used. For all scales, composite scores were calculated. In this analysis, the unidimensional use of GASA was employed and the sample was grouped by sex to detect possible sex differences. Pathological gaming and mental health variables (e.g., depression) measured at the same time were allowed to correlate. Path analysis allows investigation of theoretical assumptions systematically, and we wanted to validate the theoretical assumptions of the model by comparing the first model to alternative models with restrictions. Four new models were tested with one of the following restrictions: “no consequences of gaming” (M1) “no antecedents of gaming” (M2), “time equivalence” (M3) and “sex equivalence” (M4).

In the restricted models for *no consequences of gaming*, effects of gaming on the outcome variables (path a and path b) was set to zero. In the restricted models of *no antecedents of gaming*, effects of the outcome variable on gaming (path c and path d) was restricted to zero.

*Time equivalence* was restricted by setting all effects of gaming and outcome variables to be equal between all measures (path a = path b, path c = path d), restricting all effects of time. In the models with *sex equivalence*, effects of sex were not included in the analysis.

The restricted models were compared with the unrestricted model using the Satorra-Bentler chi square test (Satorra and Bentler, 2010), adjusted with maximum likelihood estimation with robust standard errors (MLR) scaling correction factor (Muthé and Muthén, 2018). If the assumptions of the restricted models were not consistent with the data, model fit would deteriorate. Hence, a significant result on the chi-square test would suggest that the unrestricted model fitted better with our data than the model with restricted assumptions. A non-significant result would indicate that the restricted model fitted the data equally as the unrestricted model (Bryant and Satorra, 2012), suggesting that the imposed restriction would be consistent with the data.

### Study 2

To test the predictions for a typology perspective, we classified gaming status into four groups: (1) Engaged gamers, (2) Problem gamers, (3) Addicted gamers, and (4) Non-problem/non-engaged/non-addicted contrast group (hereafter denoted “contrast group”) using the Core 4 approach (Brunborg et al., 2015).

In a series of subsequent regressions models, we investigated whether gaming status predicted mental health outcomes (hereafter called “consequences”) and whether mental health outcomes predicted gaming status (hereafter called “antecedents”). As gaming status was a nominal variable, multinomial regression analysis was used to investigate the antecedents of gaming status. To examine the consequences of gaming status, gaming status was pacifier coded and used as independent variables with mental outcomes as the dependent variable. We controlled for sex, typology identification, and scores on the previous wave on the outcome measures. Both analyses were conducted in the same model, and the analysis was repeated for each outcome measure. Categories of gaming and outcome measures were first analyzed with 1 year intervals (T1–T2, T2–T3), and then in a new model investigating the effects over 2 years (T1–T3). Results of the regression analyses will be discussed according to the findings in study 1, to further investigate the identified associations between pathological gaming to mental health over time.

### Study 3

To investigate the stability and trajectories of addicted gamers, problem gamers and engaged gamers, we estimated a hidden Markov model of transition probabilities between the three typologies of gamers, and the contrast group. Hidden Markov models are used to estimate transition probabilities between categorical variables for time series. Observed values are used to estimate the underlying and unobserved Markov process, also known as a Markov chain, which rests on the assumption that the probability of a current state is dependent on the previous state (Zucchini et al., 2016; Muthén and Muthén, 2017).

## Results

### Attrition

Of the 2,055 participants, 21 were excluded due to missing items on GASA at Wave 1. Of the remaining participants, 999 participated at all waves; 256 were missing at Wave 2, 309 were missing at Wave 3, and 470 were missing at both Waves 2 and 3. In general, predictors of being missing were weak with a few exceptions. Being missing at Wave 3 was predicted by being male (*OR* = 0.52, *p* = 0.001), and by higher alcohol consumption (*OR* = 1.10, *p* = 0.01). Being missing at Waves 2 and 3 was also predicted by being male (*OR* = 0.31, *p* = 0.00), and higher alcohol consumption (*OR* = 1.08, *p* = 0.01), and also by being addicted gamer (*OR* = 4.58, *p* = 0.02).

### Study 1

#### Antecedents and consequences of pathological gaming

The results of the association between pathological gaming and health outcomes in the unrestricted model are reported in Table 1.

**Table 1:** A cross-lagged path model of the antecedents and consequences of gaming problems.

	**Standardized beta**				**Model fit**					
	**Path A**	**Path B**	**Path C**	**Path D**	**χ^2^ (df = 8)**	**CFI**	**TLI**	**RMSEA**	**SRMR**	***n***
**DEPRESSION**										
Boys	0.14^**^	0.07	0.03	0.01	115.33	0.907	0.674	0.114	0.050	963
Girls	0.13^***^	0.12^**^	0.11^**^	0.12^**^						1,088
**ANXIETY**										
Boys	0.11^*^	0.07	0.03	−0.02	93.93	0.936	0.776	0.102	0.042	963
Girls	0.07^*^	0.07^*^	0.05	0.05						1,088
**LONELINESS**										
Boys	0.07	0.05	0.04	0.01	92.47	0.933	0.767	0.101	0.044	962
Girls	0.07	0.08	0.10^*^	0.08^*^						1,088
**ALCOHOL**										
Boys	−0.03	−0.05	−0.05	−0.06	76.62	0.938	0.784	0.091	0.038	963
Girls	0.01	0.01	−0.001	−0.04						1,087
**VERBAL AGGRESSION**										
Boys	0.09^*^	0.02	0.04	−0.04	103.73	0.923	0.730	0.108	0.043	963
Girls	0.03	0.03	0.02	−0.003						1,088
**PHYSICAL AGGRESSION**										
Boys	0.05	−0.03	0.05	0.05	87.91	0.938	0.782	0.099	0.040	963
Girls	0.04	0.06	0.08^*^	0.05						1,088

*Mental health as consequences of gaming were tested in path A and path B, while mental health as antecedents of gaming were tested in path C and path D*.

* *p < 0.05*.

** *p < 0.01*.

*** *p < 0.001*.

Results of the Satorra-Bentler test of chi square differences between an unrestricted *path model* and the restricted models (see Appendix A–D for tables) are reported in Table 2. Table 2 shows that the test for *no consequences of gaming* was significant for depression, anxiety and loneliness, indicating that the assumption of these variables not being consequences of gaming pathology were invalid. The test for *no antecedents of gaming* was significant for depression, loneliness and physical aggression, indicating that these variables are antecedents to gaming pathology. Thus, the model fit was significantly worse when restricting the consequences and antecedents in the aforementioned variables. The findings indicate that the theoretical assumption of e.g., depression not to be a consequence of pathological gaming was invalidated. Hence, we identified physical aggression to be an antecedent of pathological gaming, anxiety as a consequence of pathological gaming, and we identified a cross-lagged association between depression, loneliness and pathological gaming. The omnibus test did not reveal any sex or age differences across the three time points. This indicated that mental health antecedents and consequences of pathological gaming were the same for boys and girls, independent of age in this study.

**Table 2:** Satorra-Bentler chi square test comparing the restricted path models regarding consequences of pathological gaming, antecedents for pathological gaming, stationary assumptions, and sex differences to the unrestricted model.

	**M1: No consequences of gaming (df = 4)**	**M2: No antecedents of gaming (df = 4)**	**M3: Time equality (df = 8)**	**M4: Sex equality (df = 8)**
Depression	37.84^*^	20.47^*^	6.16	11.52
Anxiety	19.51^*^	6.18	13.27	5.99
Loneliness	12.82^*^	16.92^*^	8.50	10.43
Alcohol	2.18	5.41	12.14	13.27
Verbal aggression	7.28	2.38	8.86	7.86
Physical aggression	7.31	10.66^*^	12.03	7.44

*Degrees of freedom reported is the difference between the restricted model and the unrestricted model*.

* *p < 0.05*.

### Study 2

#### Antecedents and consequences of the three typologies of gamers

Table 3 shows the results from the multinomial regression analysis. The data indicated that depression predicted video game engagement from T1–T2, and from T1–T3. Depression also predicted problem gaming with a 1 year interval (T1–T2, T2–T3) but not over 2 years (T1–T3). Loneliness predicted video game engagement and problem gaming from T1–T2, and all categories of gaming from T1–T3. Less alcohol consumption predicted problem gaming from T2–T3, and T1–T3, whereas higher alcohol consumption predicted addicted gaming in the second year of measurement (T2–T3).Verbal aggression predicted video game engagement and problem gaming between the first two measures (T1–T2), and physical aggression predicted all categories of gaming at the same time of measurement.

**Table 3:** Multinomial regression analysis showing antecedents for “engaged gamer,” “problem gamer” and “addicted gamer.” The contrast group comprises the reference category.

	***OR*** **[95% CI]**		
	**Engaged**	**Problem**	**Addicted**
**DEPRESSION**			
T1–T2	1.11^*^ [1.02–1.22]	1.11^**^ [1.03–1.19]	1.08 [0.94–1.23]
T2–T3	1.04 [0.94–1.16]	1.11^*^ [1.02–1.21]	1.22 [0.95–1.55]
T1–T3	1.15^**^ [1.05–1.27]	1.05 [0.97–1.14]	1.09 [0.87–1.37]
**ANXIETY**			
T1–T2	1.08 [0.98–1.18]	1.05 [0.99–1.12]	1.07 [0.92–1.23]
T2–T3	1.09 [0.97–1.22]	0.98 [0.89–1.07]	0.93 [0.78–1.11]
T1–T3	1.06 [0.95–1.20]	1.05 [0.98–1.13]	0.97 [0.82–1.14]
**LONELINESS**			
T1–T2	1.11^**^ [1.04–1.19]	1.07^*^ [1.01–1.13]	1.06 [0.98–1.15]
T2–T3	1.08 [0.99–1.16]	1.05 [0.99–1.11]	1.07 [0.94–1.23]
T1–T3	1.08^*^ [1.01–1.16]	1.08^*^ [1.01–1.15]	1.16^**^ [1.05–1.28]
**ALCOHOL CONSUMPTION**			
T1–T2	0.90 [0.76–1.06]	0.97 [0.87–1.08]	1.19 [0.89–1.58]
T2–T3	0.87 [0.68–1.10]	0.78^*^ [0.63–0.98]	1.46^*^ [1.03–2.07]
T1–T3	0.94 [0.76–1.16]	0.78^**^ [0.65–0.93]	0.96 [0.75–1.23]
**VERBAL AGGRESSION**			
T1–T2	1.16^*^ [1.03– 1.31]	1.11^*^ [1.01–1.21]	1.15 [0.99–1.34]
T2–T3	1.00 [0.85– 1.16]	0.95 [0.83–1.09]	0.66 [0.41–1.08]
T1–T3	0.96 [0.84–1.10]	1.04 [0.93–1.17]	0.75 [0.54–1.04]
**PHYSICAL AGGRESSION**			
T1–T2	1.12^**^ [1.04–1.21]	1.10^**^ [1.03–1.16]	1.19^**^ [1.07–1.31]
T2–T3	1.01 [0.90–1.14]	1.05 [0.96–1.15]	0.91 [0.68–1.21]
T1–T3	1.04 [0.95–1.13]	1.02 [0.95–1.09]	0.94 [0.78–1.13]

*Gender, former level of gaming category, and former level of outcome variable is controlled for in all analyses. The indication of time (e.g., T1–T2) found under the outcome variable, indicates that the outcome variable in the first wave predicts the gaming category in the second wave*.

* *p < 0.05*,

** *p < 0.01*.

Table 4 presents the result of the linear regression analysis showing the consequences of all gaming categories compared to the contrast group. Depression was found to be a consequence of problem gaming after 1 year (T1–T2), and all categories of gaming over 2 years (T1–T3). Loneliness was found to be a consequence of problem gaming after 1 year (T1–T2), and after 2 years (T1–T3). Anxiety was found as a consequence of addicted gaming after 2 years (T1–T3). Verbal aggression was found as a consequence of gaming problems after 1 year (T1–T2).

**Table 4:** Regression analysis showing consequences of being “engaged gamer,” “problem gamer” and “addicted gamer,” compared to the contrast group with 1 year intervals between the measures.

	**STANDARDIZED REGRESSION COEFFICIENTS (STDY)**		
	**T1–T2**	**T2–T3**	**T1–T3**
**DEPRESSION**			
Engaged	0.13	0.30	0.38^*^
Problem	0.42^***^	0.12	0.33^***^
Addicted	0.52	0.33	0.58^**^
**ANXIETY**			
Engaged	0.04	0.07	0.19
Problem	0.15	0.15	0.13
Addicted	0.24	−0.04	0.38^**^
**LONELINESS**			
Engaged	0.15	0.29	0.06
Problem	0.30^**^	0.14	0.30^**^
Addicted	0.11	−0.06	0.08
**ALCOHOL CONSUMPTION**			
Engaged	−0.07	0.26	−0.02
Problem	−0.01	−0.22	−0.02
Addicted	−0.08	−0.26	−0.01
**VERBAL AGGRESSION**			
Engaged	0.10	0.01	0.25
Problem	0.19^*^	−0.03	0.11
Addicted	0.21	0.08	−0.15
**PHYSICAL AGGRESSION**			
Engaged	0.09	−0.04	0.11
Problem	0.06	−0.01	0.13
Addicted	−0.18	0.38	−0.12

*Gender, former level of gaming category, and former level of outcome variable is controlled for in all analyses*.

* *p < 0.05*,

** p < 0.01,

*** *p < 0.001*.

### Study 3

#### Stability and transitions occurring between the different typologies of gamers

**Figure 2:**
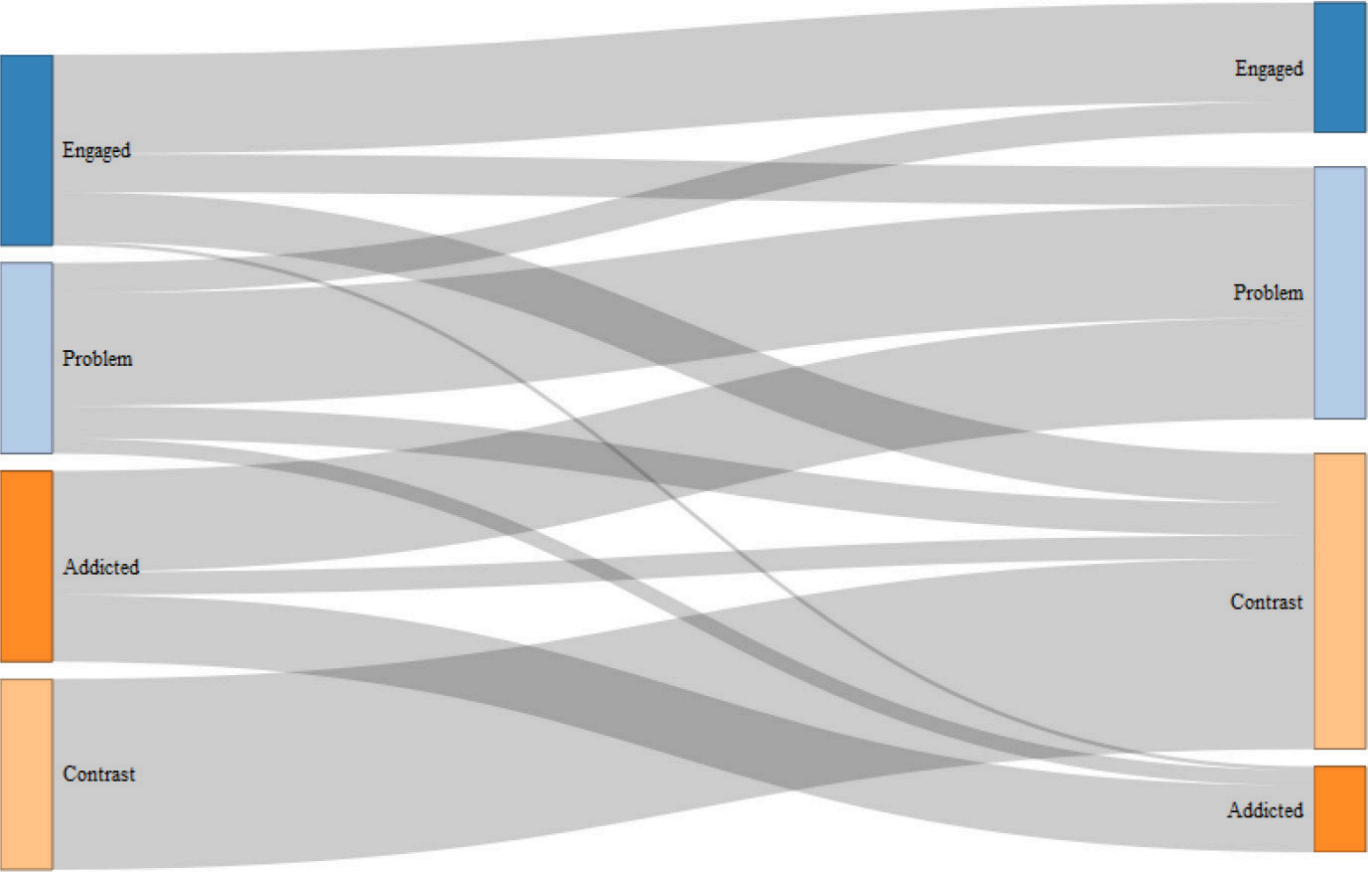
Sankey chart depicting the estimated transitions between the three typologies of gamers, and the contrast group of the adolescents that did not fall into the three gaming typologies. The estimation is based on transitions between T1–T2–T3.

The distribution of “engaged gamer,” “problem gamer,” “addicted gamer” and contrast group over the three waves is found in Appendix E, and the mean scores for each group on the outcome variable in the first and last wave are found in Appendix F. The results of the hidden Markov analysis are reported in Table 5. Figure 2 presents a Sankey chart of the estimated transition between the gamers. The stability of the addicted gamer typology was estimated to be 35%. For all typologies of gamers, except addicted, remaining in the same category over a time span of 2 years had a higher probability than changing typology. For addicted gamers there was a higher probability to transit to “problem gamer” over time (53%), than to remain in the addicted category (35%). There were virtually no transitions between “addicted” to “engaged” gamer (0%) and from “engaged” to “addicted” gamer (2%).

**Table 5:** Latent transition probability of the four categories of gamers based on a Hidden Markov analysis reported in percentage.

	**%**			
	**Engaged**	**Problem**	**Addiction**	**Contrast**
Engaged	52	20	02	26
Problem	16	59	08	17
Addiction	00	53	35	12
Contrast	00	00	00	100

## Discussion

### Study 1

#### Antecedents and consequences of pathological gaming

The aim of study 1 was to identify antecedents and consequences of pathological gaming over a timespan of 2 years. We identified, as expected, cross-lagged association between mental health symptoms in terms of loneliness and depression to pathological gaming. Regarding loneliness, our findings were consistent with the findings reported by Lemmens et al. (2011a) which indicate that loneliness might lead to pathological gaming and vice versa. In previous longitudinal studies, depression has only been found as a consequence of pathological gaming (Mößle and Rehbein, 2013; Mihara and Higuchi, 2017), but the current study indicates that symptoms of depression might predict pathological gaming too. Consistent with previous findings (Gentile et al., 2011), anxiety was identified as a consequence of pathological gaming. This might be explained by elevated symptoms in offline situations caused by less real life socialization (Lo et al., 2005), or discrepancy between online and offline identity causing insecurity in the real world. Previous research have identified physical aggression as a consequence of gaming (Lemmens et al., 2011b). In contrast, the current study identified physical aggression as an antecedent for pathological gaming. This might be caused by endogenous inclination toward physical aggression, which might be more easily gratified in video games than in the real world (Kim et al., 2008). This finding might also reflect that physical aggression can be an indication of problematic face to face relationships, making online social interaction (e.g., gaming) a more rewarding arena for aggressive youth.

Consistent with previous studies (Lemmens et al., 2011a; Brunborg et al., 2013), associations between mental health and pathological gaming in this study were found to be invariant across sex. This finding indicate that although males are more at risk of developing gaming addiction (Mentzoni et al., 2011; van Rooij et al., 2014), the way in which mental health factors function as antecedents and consequences of pathological gaming, is equal for males and females. Variation across time was not found to be a theoretical sound assumption in the current study, indicating that age differences between 17.5 and 19.5 years is not relevant for the association between mental health and pathological gaming.

### Study 2

#### Antecedents and consequences of gaming typologies

The aim of study 2 was to investigate the specific associations between mental health and the three typologies of gaming, using non-engaged/non-problem/non-addicted gamers, as the reference. We investigated typologies both as consequences and as antecedents of mental health. We expected to find that the addicted group of gamers would be associated with a higher number of antecedences, and a higher number of consequences than the other groups of gamers, which proved not to be the case in the current study. Building on the findings from study 1, the relevant results from study 2 include depression as a antecedent for engaged and problem gamer, loneliness and physical aggression as antecedents for all typologies. Regarding consequences, the relevant associations include depression as a consequence of all typologies, anxiety as a consequence of gaming addiction, and loneliness as a consequence of problem gaming typology.

As discussed in study 1, we identified cross-lagged effects between pathological gaming and loneliness and depression. When investigating the same variables in association to the typologies, loneliness was identified to be an antecedent and a consequence of problem gaming', and depression was found to be both an antecedent and a consequence of problem gaming as well as engaged gaming. The weak but significant, reciprocal association between depression and engaged gaming was somewhat surprising as previous research have not reported any detrimental effects of video game engagement (Brunborg et al., 2013). The current finding suggests that salience, tolerance, and mood modification (periphery criterias), which are symptoms of IGD (American Psychiatric Association, 2013), might be important to understand the seemingly mutual upholding association between pathological gaming and loneliness/depression found in study 1.

There were also some interesting findings specific to addicted gaming, which might explain why more severe psychopathology is found to be associated with this group (Loton et al., 2016). Anxiety was only found as a consequence for addicted gamers. Building on findings from study 1, it is notable that the identified association between anxiety and “pathological gaming” only seems to apply to “addicted gaming” in study 2. We did not find any support for an association between alcohol consumption and pathological gaming in study 1. However, investigation of the typologies revealed that high alcohol consumption predicted video game addiction, while low alcohol consumption predicted problem gaming in study 2. These findings in opposite directions might cancel out any possible effect when conceptualizing pathological gaming as unidimensional construct, and are therefore worth noticing. These findings are some of the most noticeable differences between addicted gamers and the other typologies, and might explain why video game addiction seems to cause more serious health complaints than problematic gaming (Brunborg et al., 2013). No specific reciprocal associations to addicted gaming were found, indicating that this condition predicts more complex psychopathology, rather than being predicted by mental health.

Our findings show that physical aggression predicted all categories of gaming compared to the reference group. This suggests that the association between gaming and aggression might be explained by adolescents with aggressive tendencies and related psychological characteristics (Kim et al., 2008), being more at risk for gaming pathology, rather than gaming being a cause of future aggression. We did also find indications of elevated levels of verbal aggression as an antecedent of engaged and problem gaming, and as a consequence of problem gaming, but these findings were neither consistent over the three waves, nor supported by the Satorra-Bentler analysis in study 1. In sum, our findings indicate that the link between aggression and gaming typologies should be examined further using, for instance, a proposed player-centered model (Ferguson et al., 2017) or gratification paradigm (Sherry et al., 2006), which take into account the agency of the player.

### Study 3

#### Stability and trajectories of gaming typologies

The aim of study 3 was to investigate the temporal stability of gaming typologies, and the transitions occurring between such typologies and the contrast group. The temporal stability of addicted gamers was estimated to be 35% which is in the middle of ranges reported by other studies (< 1–84%) (Gentile et al., 2011; van Rooij et al., 2011; Strittmatter et al., 2016; Rothmund et al., 2018). Thus, for the majority of addicted adolescents in the current study, the severity of gaming symptoms decreased over a timespan of 2 years. Parallel developmental changes in the period from 17 to 19 years (e.g., enhanced responsibility, romantic relationships and student activities) (Rothmund et al., 2018) may explain this. On the other hand, the group of stable “addicted gamers” was 35%, which is noteworthy, indicating that for a substantial group of gamers, symptoms of gaming disorder is persistent over 2 years. Furthermore, a high proportion of addicted gamers transited to the problem gaming category (53%), which is also associated with emotional and health complaints (Brunborg et al., 2013).

The findings suggest that for all typologies of gamers, except gaming addicts, there was a higher probability to remain in the same category over time than to leave. Furthermore, no one from the contrast group transitioned to any of the gaming typologies, which suggests that symptoms of pathological gaming emerge early in the developmental history. This might explain the lack of new recruiting into the groups of engaged, problem, and addicted gamers.

### General discussion and implications of the findings

The current paper applied a broad approach to investigate the natural course of gaming behavior over time, exploring directionality of associations with mental health, as well as trajectories between different gaming typologies. Applying a unidimensional and a typological conceptualization of gaming behavior in the same sample indicate that an unidimensional approach to gaming disorder (as used in study 1) without further exploration, might result in ignoring potentially important distinctions between gaming typologies. For instance, the direction between alcohol consumption and “problem gaming” (negative) and “addicted gaming” (positive) in study 2, was opposite of each other, while study 1 did not detect any association between alcohol consumption and pathological gaming. Hence, study 2 found that there are differences between the gaming typologies that could not be investigated in study 1, where pathological gaming was measured continuously. The distinctions between categories of heavily involved gamers might be important for identification of adolescents specifically in need of treatment, and for the development of interventions for prevention and treatment.

Results of study 2 and 3 combined provide knowledge of the natural course of different gaming typologies. Inspecting the trajectories of study 3 indicates that most addicted gamers (53%) transit to “problem gamers,” or remain addicted (35%). This is interesting with regards to findings in study 2, that point to several negative consequences of “problem gaming” and “addicted gaming.” In sum, the stability of addicted gamers and problematic gamers was quite high, and it seems fair to assume that many of these adolescents are in need of treatment or other kinds of support.

**Figure 3:**
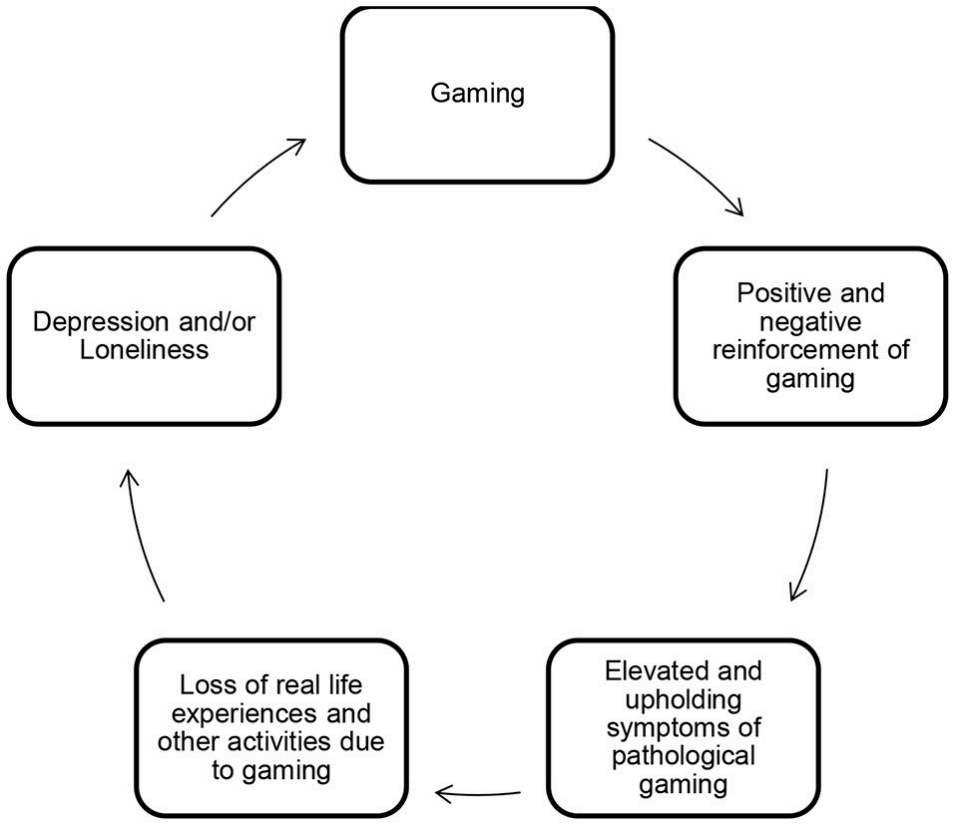
Model depicting the proposed mechanisms acting between depression, loneliness, and pathological gaming.

According to our findings in study 1, depression and loneliness seem to interact with symptoms of pathological gaming in a mutually self-enhancing and/or upholding cycle. This was by and large supported by the findings from study 2, with some distinctions between the typologies. An explanatory model of pathological internet use in adolescents (Strittmatter et al., 2016) proposes that negative offline reinforcement (e.g., escape from real life problems, negative mood, conflicts) by pathological internet use results in even more pathological internet use. Adolescents also experience positive online reinforcement (e.g., self-affirmation, identity exploration), which in turn facilitates more pathological internet use. Strittmatter et al. (2016) suggest that this cycle is more often activated and maintained by adolescents with emotional difficulties than among those without such difficulties. Further, “the displacement theory” (Gentile et al., 2017) explains how media consumption might replace important activities like sleeping and socializing. Building on this, we propose a model to explain the mutual upholding mechanisms acting between depression, loneliness, and pathological gaming in one cycle (see Figure 3). Initially, gaming might be an activity to occupy adolescents with emotional problems (Lemmens et al., 2011a). Gaming can as such provide immediate relief of an unpleasant state, as for instance agony caused by depression and/or loneliness (negative reinforcement of gaming) along with positive online experiences (positive reinforcement of gaming). Symptoms of pathological gaming might therefore be elevated and upheld by, for instance, being more salient in the adolescent life, and becoming an important strategy for mood modification. This might in turn result in loss of real life experiences and displacement of other activities (Gentile et al., 2017) and further result in increased emotional problems. Together, this might explain how adolescents with pathological gaming can struggle to escape this self-enhancing vicious cycle. To be clear, the model tries to explain the mechanisms acting between emotional problems and pathological gaming found in this study, not the magnitude of distress. The proposed model does not imply that gaming in general will lead to depression or loneliness, but rather explains the interaction with pathological gaming identified in the current study.

Findings in study 3 indicate that engaged gamers are more likely than the other typologies to transit to the contrast group, while addicted gamers are the least likely to transit to the contrast group. Furthermore, there seem to be virtually no transitions between “engaged gamers” and “addicted gamers.” This suggests that the probability becoming addicted due to engaged gaming is small. Findings in study 2 show that engaged gamers are also are the category with fewer negative consequences than the other typologies. Worth noting when inspecting the mean scores of engaged gamers (see Appendix F) is that they seem overall to have lower mean scores on all the outcome measures compared to addicted gamers typology. This might indicate that addicted gamers probably are more embedded in the proposed vicious cycle, experiencing more negative consequences of their gaming behavior and seemingly having more problems escaping it than engaged gamers.

The question of why some develop gaming disorder while other develop less severe gaming behaviors (problem or engaged gaming) remains an issue for further studies. In accordance with previous research (Lemmens et al., 2011a), study 1 found that loneliness was an antecedent for pathological gaming. Study 2 found that the association with loneliness not only was relevant for the development of gaming addiction, but also applied to problem and engaged gaming as well. Similarly, physical aggression was found to be an antecedent of all typologies, indicating that these two variables seem to predict intense gaming behavior in general. We also found differences between the typologies in study 2, suggesting that depression predicted engaged and problem gaming, but not addicted gaming, and that high alcohol consumption predicted addicted gaming, while low alcohol consumption predicted problem gaming. Future research on the differences and similarities between the typologies might help establish a greater degree of accuracy on the mechanisms that influence the development of different gaming behaviors.

### Strengths and limitations

A major strength of the present study is the broad, longitudinal approach which provides insight into the trajectories between mental health variables and pathological gaming, and specifically, enables examination of the three typologies over time. Other strengths include the large sample size, random sampling from the National Population Registry, and high initial response rate. Previous longitudinal studies have been criticized by not taking the initial level of variables into account (Scharkow et al., 2014), but in this study all analysis controlled for former level of all variables, and sex.

One limitation of the present study is the issue of generalizability. The sample consists of adolescents between the age of 17.5–19.5 years, who are the most at-risk age group for addictive behaviors, and therefore results may not generalize to other age groups. Furthermore, the attrition analysis found several predictors for dropout at T2 and T3 (sex, alcohol consumption, and addicted gaming), which might indicate a certain selection bias. This might have affected the statistical power of our study, and it could therefore have been beneficial to have a larger sample. However, the consequential control of sex, and previous level of all variables do assumingly reduce attrition effects.

Another limitation is that model fit in study 1 is not optimal, indicating that that results from study 1 should be interpreted with some caution. One explanation for the mediocre fit might be that the theoretical assumption allowing for sex differences, and inequality across time in the unrestricted model was invalid. This is supported by the results of the Satorra Bentler test. Few degrees of freedom might also inflate the RMSEA, and rejection of models with poor fit is thus not necessary recommended (Kenny et al., 2015).

Another limitation worth mentioning is that the reliability analysis showed somewhat low internal consistency (Nunnally, 1978) on alcohol consumption (3 items) in Wave 3, and on verbal aggression (3 items). However, alpha below the cutoff criterion of 0.70 does not necessarily imply low reliability (Cho and Kim, 2015), and an alpha of 0.60 can be considered acceptable when using short scales (Loewenthal, 2001) which contain < 10 items.

## Conclusion

The current study shows that mental health problems seem to interact closely with gaming pathology, both as antecedents and consequences over time. Several similarities were identified between engaged gamers, problem gamers and addicted gamers, and there seems to be a significant transition between the typologies, but not between addicted gamers and engaged gamers. “Engaged gamer” is associated with less severity in regard of negative consequences, whereas being an addicted gamer is associated with more severe psychopathology. Viewing gaming problems from a typological perspective might be useful in further assessment and conceptualization of video game addiction, both in research and in clinical settings. Further, the results suggest that gaming disorder has a relatively high stability, indicating that for a substantial group addicted gamers, their symptoms do not seem to resolve spontaneously, indicating the need for intervention or clinical management.

## Author contributions

SP, RM, TT, HM, and DK stood for the conception and design of the work. All authors contributed to the acquisition, analysis, and interpretation of data. EK drafted the work. All authors revised the work critically in terms of important intellectual content. All authors approved the final version and are accountable for all aspects of the work in terms of ensuring that questions related to the accuracy or integrity of any part of the work were appropriately investigated and resolved.

### Conflict of interest statement

The authors declare that the research was conducted in the absence of any commercial or financial relationships that could be construed as a potential conflict of interest.
