# Supplementary material for: A Cross-Lagged Study of Developmental Trajectories of Video Game Engagement, Addiction, and Mental Health
Source: Front Psychol. 2018 Nov 21;9:2239. doi: 10.3389/fpsyg.2018.02239 (PMC6258776; doi:10.3389/fpsyg.2018.02239)
Supplement: Supplementary file 1 [file Table_1.docx]

**APPENDIX**

**Appendix A.** A cross-lagged path model of the antecedents and consequences of gaming problems restricted consequences of gaming (M1). mental health as consequences of gaming were tested in path A and path B, while mental health as antecedents of gaming were tested in path C and path D.

|  |  |  |  |  |  |  | |  |  | |  | |  | |  | |
| --- | --- | --- | --- | --- | --- | --- | --- | --- | --- | --- | --- | --- | --- | --- | --- | --- |
|  | **Standardized beta** | | | |  | **Model fit** | | | | | | | | | | |
|  |  |  |  |  |  |  |  | | |  | |  | |  | |  |
|  | Path A | Path B | Path C | Path D |  | χ^2^(df=12) | CFI | | | TLI | | RMSEA | | SRMR | | *n* |
| **Depression** |  |  |  |  |  |  |  | | |  | |  | |  | |  |
| Boys | 0.00 | 0.00 | 0.04 | 0.02 |  | 154.94 | 0.876 | | | 0.711 | | 0.108 | | 0.075 | | 963 |
| Girls | 0.00 | 0.00 | 0.11** | 0.13** |  |  |  | | |  | |  | |  | | 1088 |
| **Anxiety** |  |  |  |  |  |  |  | | |  | |  | |  | |  |
| Boys | 0.00 | 0.00 | 0.03 | -0.01 |  | 118.78 | 0.920 | | | 0.814 | | 0.093 | | 0.056 | | 963 |
| Girls | 0.00 | 0.00 | 0.052 | 0.052 |  |  |  | | |  | |  | |  | | 1088 |
| **Loneliness** |  |  |  |  |  |  |  | | |  | |  | |  | |  |
| Boys | 0.00 | 0.00 | 0.04 | 0.01 |  | 110.32 | 0.922 | | | 0.819 | | 0.089 | | 0.056 | | 962 |
| Girls | 0.00 | 0.00 | 0.10* | 0.08* |  |  |  | | |  | |  | |  | | 1088 |
| **Alcohol** |  |  |  |  |  |  |  | | |  | |  | |  | |  |
| Boys | 0.00 | 0.00 | -0.05 | -0.06 |  | 82.83 | 0.936 | | | 0.851 | | 0.076 | | 0.039 | | 963 |
| Girls | 0.00 | 0.00 | -0.001 | -0.04 |  |  |  | | |  | |  | |  | | 1087 |
| **Verbal aggression** |  |  |  |  |  |  |  | | |  | |  | |  | |  |
| Boys | 0.00 | 0.00 | 0.04 | -0.04 |  | 120.04 | 0.913 | | | 0.797 | | 0.094 | | 0.048 | | 963 |
| Girls | 0.00 | 0.00 | 0.02 | -0.002 |  |  |  | | |  | |  | |  | | 1088 |
| **Physical aggression** |  |  |  |  |  |  |  | | |  | |  | |  | |  |
| Boys | 0.00 | 0.00 | 0.05 | 0.04 |  | 103.64 | 0.929 | | | 0.833 | | 0.086 | | 0.045 | | 963 |
| Girls | 0.00 | 0.00 | 0.08* | 0.05 |  |  |  | | |  | |  | |  | | 1088 |
| **p* < 0.05. ** *p* < 0.01. *** *p* < 0.001. | | | | | | | | | | | | | | | | |

**Appendix B.** A cross lagged path model of the antecedents and consequences of gaming problems restricted on antecedents of gaming (M2). Mental health as consequences of gaming was tested in path A and path B, while mental health as antecedents of gaming was tested in path C and path D.

|  |  |  |  |  |  |  | |  | | |  |  | |  | |  |
| --- | --- | --- | --- | --- | --- | --- | --- | --- | --- | --- | --- | --- | --- | --- | --- | --- |
|  | **Standardized beta** | | | |  | **Model fit** | | | | | | | | | | |
|  |  |  |  |  |  |  |  | |  |  | | |  | |  | |
|  | Path A | Path B | Path C | Path D |  | χ^2^(df=12) | CFI | | TLI | RMSEA | | | SRMR | | *n* | |
| **Depression** |  |  |  |  |  |  |  | |  |  | | |  | |  | |
| Boys | 0.15*** | 0.07 | 0.00 | 0.00 |  | 137.31 | 0.891 | | 0.747 | 0.101 | | | 0.062 | | 963 | |
| Girls | 0.13*** | 0.13*** | 0.00 | 0.00 |  |  |  | |  |  | | |  | | 1088 | |
| **Anxiety** |  |  |  |  |  |  |  | |  |  | | |  | |  | |
| Boys | 0.11** | 0.07 | 0.00 | 0.00 |  | 109.15 | 0.928 | | 0.831 | 0.089 | | | 0.046 | | 963 | |
| Girls | 0.07* | 0.07* | 0.00 | 0.00 |  |  |  | |  |  | | |  | | 1088 | |
| **Loneliness** |  |  |  |  |  |  |  | |  |  | | |  | |  | |
| Boys | 0.08 | 0.05 | 0.00 | 0.00 |  | 116.64 | 0.917 | | 0.807 | 0.092 | | | 0.056 | | 962 | |
| Girls | 0.07 | 0.09 | 0.00 | 0.00 |  |  |  | |  |  | | |  | | 1088 | |
| **Alcohol** |  |  |  |  |  |  |  | |  |  | | |  | |  | |
| Boys | -0.03 | -0.06 | 0.00 | 0.00 |  | 86.10 | 0.933 | | 0.844 | 0.078 | | | 0.042 | | 963 | |
| Girls | 0.01 | 0.01 | 0.00 | 0.00 |  |  |  | |  |  | | |  | | 1087 | |
| **Verbal aggression** |  |  |  |  |  |  |  | |  |  | | |  | |  | |
| Boys | 0.09* | 0.02 | 0.00 | 0.00 |  | 117.10 | 0.915 | | 0.803 | 0.092 | | | 0.043 | | 963 | |
| Girls | 0.03 | 0.03 | 0.00 | 0.00 |  |  |  | |  |  | | |  | | 1088 | |
| **Physical aggression** |  |  |  |  |  |  |  | |  |  | | |  | |  | |
| Boys | 0.05 | -0.03 | 0.00 | 0.00 |  | 107.73 | 0.925 | | 0.826 | 0.088 | | | 0.049 | | 963 | |
| Girls | 0.04 | 0.06 | 0.00 | 0.00 |  |  |  | |  |  | | |  | | 1088 | |
| *p < 0.05. ** p < 0.01. *** p < 0.001. | | | | | | | | | | | | | | | | |

|  |  |  |  |  |  |  |  | | |  | | |  | | |  | |  | | | |
| --- | --- | --- | --- | --- | --- | --- | --- | --- | --- | --- | --- | --- | --- | --- | --- | --- | --- | --- | --- | --- | --- |
|  | **Standardized beta** | | | |  | **Model fit** | | | | | | | | | | | | | | | |
|  |  |  |  |  |  |  | |  | | |  | | |  | | |  | | |  | |
|  | Path A | Path B | Path C | Path D |  | χ^2^(df=16) | | | CFI | | | TLI | | | RMSEA | | | | SRMR | | *n* |
| **Depression** |  |  |  |  |  |  | | |  | | |  | | |  | | | |  | |  |
| Boys | 0.11*** | 0.11*** | 0.02 | 0.02 |  | 117.03 | | | 0.912 | | | 0.847 | | | 0.078 | | | | 0.055 | | 963 |
| Girls | 0.13*** | 0.13*** | 0.11*** | 0.12*** |  |  | | |  | | |  | | |  | | | |  | | 1088 |
| **Anxiety** |  |  |  |  |  |  | | |  | | |  | | |  | | | |  | |  |
| Boys | 0.09** | 0.09** | 0.01 | 0.01 |  | 100.58 | | | 0.937 | | | 0.890 | | | 0.072 | | | | 0.048 | | 963 |
| Girls | 0.07*** | 0.07*** | 0.05** | 0.05** |  |  | | |  | | |  | | |  | | | |  | | 1088 |
| **Loneliness** |  |  |  |  |  |  | | |  | | |  | | |  | | | |  | |  |
| Boys | 0.07* | 0.07* | 0.02 | 0.02 |  | 105.48 | | | 0.929 | | | 0.876 | | | 0.074 | | | | 0.052 | | 962 |
| Girls | 0.08** | 0.07** | 0.08*** | 0.09*** |  |  | | |  | | |  | | |  | | | |  | | 1088 |
| **Alcohol** |  |  |  |  |  |  | | |  | | |  | | |  | | | |  | |  |
| Boys | -0.04 | -0.05 | -0.06* | -0.06* |  | 86.63 | | | 0.936 | | | 0.889 | | | 0.066 | | | | 0.057 | | 963 |
| Girls | 0.01 | 0.01 | -0.02 | -0.02 |  |  | | |  | | |  | | |  | | | |  | | 1087 |
| **Verbal aggression** |  |  |  |  |  |  | | |  | | |  | | |  | | | |  | |  |
| Boys | 0.05* | 0.06* | 0.003 | 0.003 |  | 111.05 | | | 0.924 | | | 0.866 | | | 0.076 | | | | 0.049 | | 963 |
| Girls | 0.03 | 0.03 | 0.01 | 0.01 |  |  | | |  | | |  | | |  | | | |  | | 1088 |
| **Physical aggression** |  |  |  |  |  |  | | |  | | |  | | |  | | | |  | |  |
| Boys | 0.01 | 0.01 | 0.05 | 0.04 |  | 97.10 | | | 0.937 | | | 0.889 | | | 0.070 | | | | 0.048 | | 963 |
| Girls | 0.05** | 0.05** | 0.06** | 0.06** |  |  | | |  | | |  | | |  | | | |  | | 1088 |
| *p < 0.05. ** p < 0.01. *** p < 0.001. | | | | | | | | | | | | | | | | | | | | | |

**Appendix C.** A cross lagged path model of the antecedents and consequences of gaming problems with time equivalence (M3). Mental health as consequences of gaming was tested in path A and path B, while mental health as antecedents of gaming was tested in path C and path D.

|  |  |  |  |  |  |  |  | |  |  | |  | |  | | |
| --- | --- | --- | --- | --- | --- | --- | --- | --- | --- | --- | --- | --- | --- | --- | --- | --- |
|  | **Standardized beta** | | | |  | **Model fit** | | | | | | | | | | |
|  |  |  |  |  |  |  | |  | | |  | |  | |  |  |
|  | Path A | Path B | Path C | Path D |  | χ^2^(df=16) | | CFI | | | TLI | | RMSEA | | SRMR | *n* |
| **Depression** |  |  |  |  |  |  | |  | | |  | |  | |  |  |
| Boys | 0.16*** | 0.11** | 0.06** | 0.06* |  | 120.33 | | 0.910 | | | 0.842 | | 0.080 | | 0.060 | 963 |
| Girls | 0.10*** | 0.08** | 0.09** | 0.10** |  |  | |  | | |  | |  | |  | 1088 |
| **Anxiety** |  |  |  |  |  |  | |  | | |  | |  | |  |  |
| Boys | 0.10** | 0.07* | 0.03 | 0.02 |  | 107.07 | | 0.932 | | | 0.881 | | 0.075 | | 0.058 | 963 |
| Girls | 0.06** | 0.05* | 0.05 | 0.04 |  |  | |  | | |  | |  | |  | 1088 |
| **Loneliness** |  |  |  |  |  |  | |  | | |  | |  | |  |  |
| Boys | 0.08* | 0.08* | 0.06** | 0.04* |  | 102.00 | | 0.932 | | | 0.881 | | 0.072 | | 0.054 | 962 |
| Girls | 0.05* | 0.05* | 0.09** | 0.07* |  |  | |  | | |  | |  | |  | 1088 |
| **Alcohol** |  |  |  |  |  |  | |  | | |  | |  | |  |  |
| Boys | -0.01 | -0.02 | -0.02 | -0.04 |  | 84.96 | | 0.938 | | | 0.891 | | 0.065 | | 0.056 | 963 |
| Girls | -0.01 | -0.02 | -0.02 | -0.05 |  |  | |  | | |  | |  | |  | 1087 |
| **Verbal aggression** |  |  |  |  |  |  | |  | | |  | |  | |  |  |
| Boys | 0.06* | 0.03 | 0.02 | -0.01 |  | 109.65 | | 0.925 | | | 0.868 | | 0.076 | | 0.055 | 963 |
| Girls | 0.04* | 0.02 | 0.02 | -0.01 |  |  | |  | | |  | |  | |  | 1088 |
| **Physical aggression** |  |  |  |  |  |  | |  | | |  | |  | |  |  |
| Boys | 0.05 | 0.02 | 0.05* | 0.04 |  | 101.98 | | 0.933 | | | 0.883 | | 0.072 | | 0.057 | 963 |
| Girls | 0.04 | 0.02 | 0.07* | 0.05 |  |  | |  | | |  | |  | |  | 1088 |
| *p < 0.05. ** p < 0.01. *** p < 0.001. | | | | | | | | | | | | | | | | |

**Appendix D.** A cross lagged path model of the antecedents and consequences of gaming problems with sex equivalence (M4). Mental health as consequences of gaming was tested in path A and path B, while mental health as antecedents of gaming was tested in path C and path D.

**Appendix E.** Table showing the distribution of the three typologies of gamers and the contrast group over time (T1-T3).

|  | T1 | T2 | T3 |
| --- | --- | --- | --- |
| **Engaged** | **85** | **48** | **39** |
| Female | 19 | 12 | 11 |
| Male | 66 | 36 | 28 |
| **Problem** | **210** | **116** | **73** |
| Female | 42 | 31 | 20 |
| Male | 169 | 85 | 53 |
| **Addicted** | **45** | **25** | **13** |
| Female | 9 | 4 | 3 |
| Male | 36 | 21 | 10 |
| **Contrast** | **1694** | **1132** | **1140** |
| Female | 1010 | 727 | 749 |
| Male | 680 | 403 | 388 |
| Total | 2034 | 1321 | 1265 |

**Appendix F.** Table showing the mean scores of the outcome variables measured at T1 and T3. Gaming typology and outcome variable is measured at the same wave (e.g. Engagement T1 on Anxiety T1).

|  | Engaged gamers | |  | Problem gamers | |  | Addicted gamers | |  | Contrast group | |
| --- | --- | --- | --- | --- | --- | --- | --- | --- | --- | --- | --- |
|  | T1 | T3 |  | T1 | T3 |  | T1 | T3 |  | T1 | T3 |
| Anxiety | 6,3 (n=84) | 6,7 (n=38) |  | 5,7 (n=205) | 6,3 (n=71) |  | 6,7 (n=43) | 8,0 (n=13) |  | 5,5 (n=1671) | 5,2 (n=119) |
| Depression | 4,8 (n=84) | 4,6 (n=39) |  | 4,7 (n=207) | 4,7 (n=71) |  | 6,1 (n=45) | 7,9 (n=13) |  | 3,4 (n=1668) | 2,8 (n=1124) |
| Loneliness | 5,9 (n=83) | 7,5 (n=39) |  | 6,2 (n=203) | 6,9 (n=69) |  | 7,9 (n=44) | 8,3 (n=13) |  | 4,6 (n =1654) | 4,9 (n=1113) |
| Verbal aggression | 6,4 (n=84) | 6,1 (n=39) |  | 7,1 (n=208) | 6,3 (n=73) |  | 7,8 (44) | 7,2 (n=13) |  | 6,1 (n=1680) | 5,7 (n=1135) |
| Physical aggression | 7,5 (n=84) | 6,8 (n=39) |  | 8,4 (n=208) | 6,7 (n=72) |  | 9,8 (n=44) | 8,6 (n=12) |  | 6,5 (n=1682) | 5,7 (n=1135) |
| Alcohol | 6,9 ( n=56) | 8,4 (n=29) |  | 7,3 (n=150) | 7,3 (n=66) |  | 7,5 (n=32) | 8,7 (n=10) |  | 6,8 (n=1203) | 7,7 (n=989) |

**Appendix G.** Table showing the correlations between the outcome measures at T1.

| Measure |  | 1. | 2. | 3. | 4. | 5. | 6. |
| --- | --- | --- | --- | --- | --- | --- | --- |
|  |  |  |  |  |  |  |  |
| 1.Depression |  | - |  |  |  |  |  |
| 2.Anxiety |  | .48** | - |  |  |  |  |
| 3.Loneliness |  | .48** | .38** | - |  |  |  |
| 4.Alcohol |  | .07** | .04 | -.07* | - |  |  |
| 5.Verbal Aggression |  | .24** | .28** | .19** | .15** | - |  |
| 6. Physical Aggression |  | .29** | .27** | .20** | .26** | .57** | - |
|  |  |  |  |  |  |  |  |
| *p < 0.05. ** p < 0.01. | | | | | | | |
|  |  |  |  |  |  |  |  |
